# Metastatic melanoma to the gallbladder presented as a polyp with acute cholecystitis: A case report from Syria

**DOI:** 10.1016/j.amsu.2022.103514

**Published:** 2022-03-28

**Authors:** Moatasem Hussein Al-janabi, Jollanar Ghanem Mohammad, Aya Yaser Mohsen, Ahmad Saad, Rana Issa

**Affiliations:** a Department of Pathology, Cancer Research Center, Tishreen University Hospital, Lattakia, Syria; b Tishreen University Faculty of Medicine, Lattakia, Syria; c Department of General Surgery, Tishreen University Hospital, Lattakia, Syria; d Department of Pathology, Tishreen University Hospital, Lattakia, Syria

**Keywords:** Malignant melanoma, Polyp, Gallbladder metastases

## Abstract

**Introduction and importance:**

Malignant melanoma is one of the most aggressive unpredictable tumors that can metastasize to any organ. Metastases from cutaneous melanoma to the gallbladder are exceedingly rare. Most patients with gallbladder metastases from malignant melanoma are usually asymptomatic; therefore, its diagnosis can be a real challenge.

**Case presentation:**

We report a case of a 71-year-old woman with a past history of cutaneous melanoma, who clinically presented with signs and symptoms of acute cholecystitis. Ultrasound investigation of the gallbladder revealed intraluminal polyp. Gross inspection of the excised gallbladder showed a polypoid lesion in the fundus. Microscopically, the H&E-stained sections revealed nests of malignant cells, occasionally with pigmented cytoplasm. The diagnosis of metastatic melanoma was reported, and supported by the results of the immunohistochemical stains.

**Clinical discussion:**

Malignant melanoma is a very aggressive type of skin cancer. It arises from the melanocytes in the epidermis, uvea, meninges, and intestinal tract. Worldwide, only 40 cases of metastatic melanoma to the gallbladder were documented. Moreover, reviewing the English-language literature showed that primary melanoma is an extraordinary event and was described in about 28 cases. Clinically, metastases from cutaneous melanoma to the gallbladder are usually asymptomatic.

**Conclusion:**

We report a metastatic malignant melanoma to the gallbladder presented as a polypoid lesion and clinically manifested as acute cholecystitis.

## Highlights

• Melanoma is the most serious type of skin cancer. The gallbladder is an extremely rare location for metastatic melanoma.
• We report a case of a 71-years old woman with metastatic melanoma to the gallbladder presented as acute cholecystitis.
• Melanoma is a very aggressive tumor. Clinically, metastases from melanoma to the gallbladder are usually asymptomatic.

## 1 Introduction

Melanoma is the most serious type of skin cancer that develops from cells known as melanocytes. Although melanoma is less common than basal cell carcinoma and squamous cell carcinoma, it is more aggressive and with a higher tendency to grow locally and metastasize [1]**.** It can metastasize to subcutaneous tissue, lymph nodes, lungs, liver, bones, or brain. Gastrointestinal metastases from melanoma account for 2–4% of cases, with the most frequent sites being the small bowel (35–67%), colon (9–15%), and stomach (5–7%) [2]. The gallbladder is an extremely rare location for metastatic melanoma. In living patients, the involvement of the gallbladder by metastatic melanoma has been rarely documented, as most of the cases were asymptomatic [3]. In this report, we describe a case of metastatic melanoma to the gallbladder presented clinically as acute cholecystitis.

This case report has been reported in line with the SCARE criteria 2020 [4].

## 2 Case presentation

**Fig. 1:**
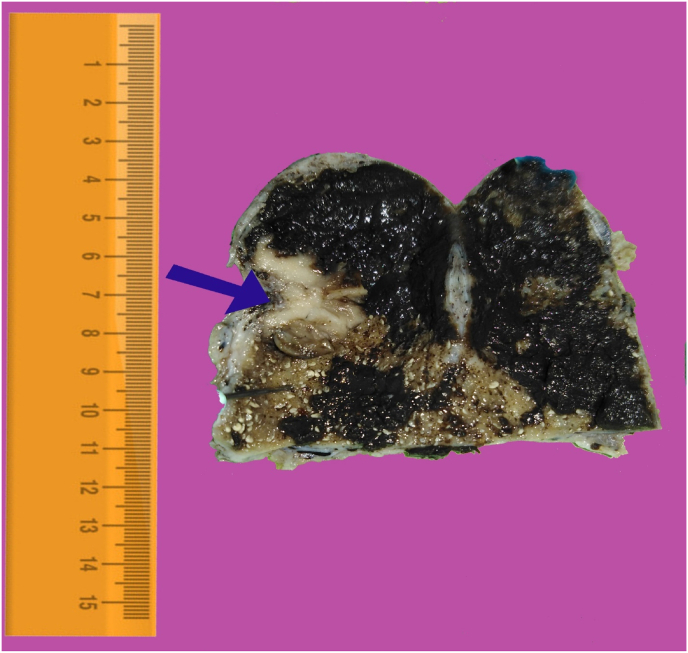
Gross image of excised gallbladder showing brownish pigmented polypoid lesion, 2.5 cm in diameter located in fundus region (blue arrow). The surrounding mucosa was black and velvet. (For interpretation of the references to color in this figure legend, the reader is referred to the Web version of this article.)

**Fig. 2:**
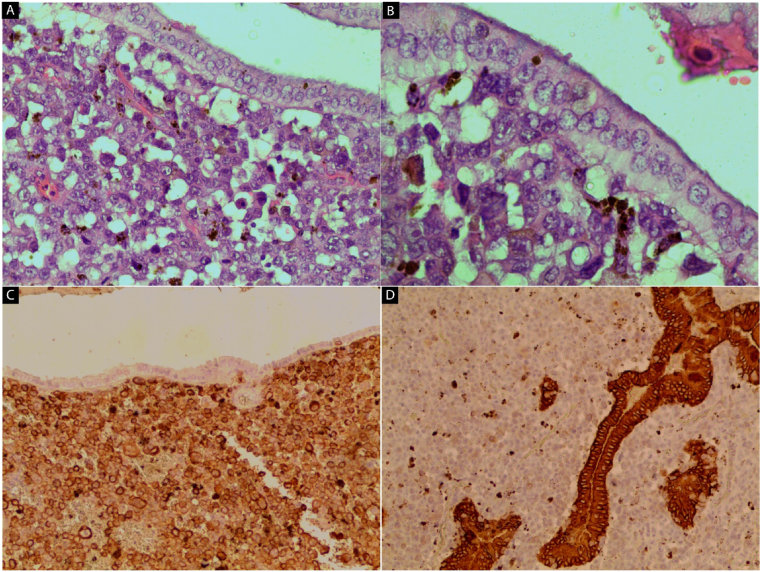
H&E stain (A and B) and IHC (C and D): Microscopic images of the gallbladder. (A) Malignant epithelioid cells in lamina propria of the gallbladder (x 40). (B) malignant cells showed large hyperchromatic atypical nuclei with prominent nucleoli and coarse brown cytoplasmic pigment (x 100). (C) The tumor cells are positive for melanoma cocktail (x 40). (D) Pan-cytokeratin (CK) is negative (x 100). (For interpretation of the references to color in this figure legend, the reader is referred to the Web version of this article.)

A 71-year-old woman presented to the emergency department at Tishreen University Hospital in 2021 with repeated attacks of right upper quadrant abdominal pain radiating to the back and right shoulder and worsened by fatty meals. The patient was a non-smoker and non-alcoholic. She did not use any specific drugs and had no history of allergy. She underwent surgical excision for a skin tumor located in the right deltoid region, 7 years ago. There was no tumor history in her family; particularly no history of skin tumors. On physical examination, there was tenderness in the right upper quadrant with a positive Murphy's sign. Laboratory tests showed an elevation of white blood cells (26,400 mm^3^) with granulocytes (45%) and lymphocytes (54%). Other Routine blood values were within normal limits. Abdomen ultrasound showed a polypoid mass protruding in the lumen of the gallbladder and located in the fundus area. Diagnosis of acute cholecystitis was made and the laparoscopic cholecystectomy was done. The gallbladder was sent to the pathology department. The excised gallbladder measured 8 cm × 2.5 cm with a wall thickness of about 0.3 cm. The serosa was unremarkable. Inspection of the luminal aspect of the gallbladder revealed a 2.5 cm measured, white, brownish mottled polypoid lesion, located in the fundus region. The surrounding mucosa was velvet and black in color [Fig. 1]. There was necrotic mucinous material in the lumen. Microscopic examination of the polypoid lesion revealed proliferation of variably large nests of malignant epithelioid cells with large hyperchromatic nuclei and prominent nucleoli. The cytoplasm was eosinophilic with occasional coarse brown pigments [Fig. 2A] [Fig. 2B]. Our initial diagnosis on the HE stained sections was malignant melanoma. To confirm our diagnosis immunohistochemical stains were applied. The tumor cells were positive for melanoma cocktail [Fig. 2C] and were negative for pan-cytokeratin [Fig. 2D]. No molecular test was performed, as it was not available in our lab. There was no medical record for the patient in our hospital. By contacting the in-charge surgeon, we were informed that the patient had a history of cutaneous malignant melanoma, seven years ago, in the right deltoid region. That time, the tumor was excised and followed by chemotherapy with paclitaxel plus carboplatin. Our final diagnosis was metastatic malignant melanoma to the gallbladder, presented as a polypoid lesion and accompanied by acute cholecystitis. The patient was discharged 3 days later, without any complications. Four weeks later, her course was complicated by the development of increasing neurological symptoms. Unfortunately, brain metastases were found on CT scan.

## 3 Discussion

Malignant melanoma is a very aggressive type of skin cancer. It arises from the melanocytes in the epidermis, uvea, meninges, and intestinal tract [5]. Malignant melanoma often metastasizes to the skin, lungs, liver, and brain [2]. Primary and secondary melanomas of the gallbladder are extraordinary events. Worldwide, in the English-language literature, the documented cases of primary malignant melanoma were approximately 28 cases, with 40 cases of secondary involvement [6,7]. In most cases of metastatic melanoma patients were asymptomatic. But in very few cases, the leading clinical manifestations were symptoms that mimic acute cholecystitis, and that was the main complaint of our patient [8,9]. In both symptomatic and asymptomatic cases, abdominal ultrasound examination is the imaging study of choice for the evaluation of gallbladder metastases [8,10]. Within the gallbladder, the metastatic melanoma tends to appear as a flat infiltrative lesion or as a polypoid tumor, like in our case. Despite the lack of standard guidelines for the treatment of metastatic melanoma, surgical resection remains the procedure of choice in case of resectable lesions [10,11].

## 4 Conclusion

Malignant melanoma is one of the most aggressive malignant tumors of the skin, and it is characterized by high mortality. Metastases from malignant melanoma to the gallbladder are exceptional events. Patients with a history of malignant melanoma should be closely followed up in order to detect metastasis. Any abdominal complaint should be further investigated with imaging studies to exclude secondary involvement by malignant melanoma.

## Ethical Approval

No ethical approval was needed for this case report.

## Sources of funding

None declared.

## Author contributions

Moatasem Hussein Al-janabi: study design, data collections, data analysis, and writing. Jollanar Ghanem Mohammad: study design, data analysis, and writing. Aya Yaser Mohsen: study design, data analysis, and writing. Ahmad Saad: performed this surgery and data collections. Rana Issa: in reviewing the manuscript.

## Registration of research studies

Not applicable.

## Guarantor

Rana Issa.

## Consent

Written informed consent was obtained from the patient for publication of this case report and accompanying images. A copy of the written consent is available for review by the Editor-in-Chief of this journal.

## Provenance and peer review

Not commissioned, externally peer-reviewed.

## Declaration of competing Interest

The authors have no conflicts of interest to declare.
